# Pre-, Pro-, Post- and Synbiotics in Pediatric Short Bowel Syndrome: A Narrative Review of Current Evidence

**DOI:** 10.3390/children13030349

**Published:** 2026-02-28

**Authors:** Roberta Giusy Ruiz, Annalisa Morelli, Rosangela Grieco, Sabrina Cardile, Teresa Capriati, Chiara Maria Trovato, Giulia Bolasco, Daniela Knafelz, Fiammetta Bracci, Arianna Alterio, Antonella Diamanti

**Affiliations:** 1 Digestive Diseases and Nutritional Rehabilitation Unit, Nutritional Treatments of Complex Diseases Research Unit, Bambino Gesù Children’s Hospital, Istituto di Ricovero e Cura a Carattere Scientifico (IRCCS), 00165 Rome, Italy; robertagiusy.ruiz@opbg.net (R.G.R.); rosangela.grieco@opbg.net (R.G.); sabrina.cardile@opbg.net (S.C.); teresa.capriati@opbg.net (T.C.); chiaramaria.trovato@opbg.net (C.M.T.); giulia.bolasco@opbg.net (G.B.); daniela.knafelz@opbg.net (D.K.); fiammetta.bracci@opbg.net (F.B.); arianna.alterio@opbg.net (A.A.); antonella.diamanti@opbg.net (A.D.); 2 Department of Paediatrics, Università Cattolica del Sacro Cuore, Istituto di Ricovero e Cura a Carattere Scientifico (IRCCS), 00168 Rome, Italy; 3 Department of Medicine, Surgery and Dentistry “Scuola Medica Salernitana”, Pediatrics Section, University of Salerno, 84081 Baronissi, Italy; 4 Pediatric Gastroenterology and Liver Unit, Maternal and Child Health Department, Sapienza-University of Rome, 00185 Rome, Italy

**Keywords:** short bowel syndrome (SBS), pediatric, microbiota, probiotics, synbiotics, dysbiosis

## Abstract

Background: Pediatric Short bowel syndrome (SBS) is the leading cause of intestinal failure and is characterized by persistent dysbiosis that negatively impacts intestinal adaptation and growth. Although microbiota modulation via pre-, pro-, and synbiotics represents a promising strategy, current evidence remains fragmented. This narrative review aims to critically assess the efficacy and safety of such interventions in the management of pediatric SBS. Methods: A structured literature search was conducted on PubMed up to November 2025. Fourteen relevant studies were included, comprising clinical trials, preclinical animal models, and significant case reports regarding the use of biotics in SBS. Results: The analysis reveals a microbiological dichotomy based on nutritional status: parenteral nutrition (PN)-dependent patients exhibit an excess of Proteobacteria associated with infectious risk, whereas weaned patients present a metabolic risk of D-lactic acidosis due to carbohydrate fermentation. Regarding efficacy, long-term synbiotic treatments (>12 months) demonstrated significant improvements in growth and nutritional status, likely mediated by increased production of short-chain fatty acids (SCFAs) and mucosal adaptation, unlike short-term probiotic cycles. However, serious adverse events (Lactobacillus sepsis and D-lactic acidosis) were reported, predominantly in patients with central venous catheters or malabsorption. Conclusions: Biotics offer therapeutic potential for intestinal failure, but their use cannot be empirical. The safety profile should be carefully selected, especially in central venous catheter (CVC) carriers. Future strategies must evolve towards precision medicine, prioritizing non-D-lactate-producing strains and personalized protocols based on the patient’s nutritional phase.

## 1. Introduction

### 1.1. Short Bowel Syndrome in Pediatric Age

Short bowel syndrome (SBS) in children is a complex malabsorptive disorder resulting from the loss or dysfunction of a significant portion of the small intestine, most commonly due to surgical resection, less frequently caused by congenital defects or loss of absorption capacity of the gut associated with disease. SBS is the leading cause of pediatric intestinal failure, defined as the reduction in functional gut, leading to the inability of the remaining intestine to support normal growth and development, typically requiring parenteral nutrition (PN) [1]. The clinical consequences include impaired absorption of macronutrients, micronutrients, fluids and electrolytes, often necessitating long-term PN and multidisciplinary management to prevent and address complications such as intestinal failure-associated liver disease (IFALD), catheter-related bloodstream infections (CRBIs), and small intestinal bacterial overgrowth (SIBO) [2].

Recent advances in care, including early initiation of enteral nutrition, individualized nutritional strategies, and intestinal rehabilitation programs, have improved survival and quality of life, with current pediatric SBS survival rates exceeding 90%. Ongoing research efforts continue to refine best practices for the management and long-term follow-up of children with SBS [3]. It is well established that the intestinal microbiota plays a fundamental role in the health of these patients; thus, recent research has been focusing on finding therapies to restore gut bacteria homeostasis [4].

### 1.2. Microbiota Alteration in Pediatric SBS

Children with SBS display a profoundly altered gut microbiota compared with their healthy peers, showing persistent dysbiosis characterized by reduced bacterial diversity, loss of beneficial anaerobes (Bifidobacterium, Bacteroidetes), depletion of anti-inflammatory Clostridia, and expansion of potentially pathogenic taxa, particularly Proteobacteria and Enterobacteriaceae [5].

The development of dysbiosis in SBS patients appears to stem from several interconnected factors. One of the most relevant influencers is the loss of intestinal surface area and altered anatomy which follows surgical bowel resection: removal of large portions of intestine, especially the ileocecal valve or the colon, causes a drastic decrease in the absorptive surfaces and consequent disruption of normal intestinal function, which induces changes in local intestinal environment, transit time and luminal flow, all of which contribute to the proliferation of opportunistic and potentially pathogenic bacteria (e.g., Proteobacteria) while reducing beneficial anaerobes (e.g., Firmicutes, Bacteroidetes). In fact, it has been demonstrated that children with shorter remnant bowel (<35 cm) had greater reliance on PN, impaired linear growth, and a microbiota enriched in Proteobacteria, while those with longer remnant bowel showed relatively higher proportions of Firmicutes [6,7,8,9]. Reliance on PN and reduction in enteral feeding are other key contributors to intestinal flora alterations: as PN bypasses the intestine, fewer nutrients can reach the gut lumen, thus leading to a decreased microbial diversity and encouragement of pathogenic strains such as Enterobacteriaceae [9,10]. The altered motility and stasis occurring in shortened bowel, especially in cases of absence of the ileocecal valve, predispose to SIBO, which not only worsens dysbiosis but also contributes to further malabsorption [11,12]. Finally, medications commonly used in SBS, such as antibiotics, acid-suppressing drugs and agents that alter gut motility, may additionally destabilize gut flora and promote dysbiosis [11,13].

The consequences of dysbiosis include the disruption in the production of beneficial microbial metabolites, such as short-chain fatty acids (SCFAs) and alteration of bile acid metabolism, both of which are factors contributing to mucosal integrity and intestinal adaptation [10,14].

Dysbiosis in SBS also contributes to alterations such as impaired intestinal adaptation, worsened nutrient malabsorption, prolonged dependence on PN, increased susceptibility to sepsis, a higher risk of IFALD and an increased risk of SIBO [4,13,15].

### 1.3. Pre-, Pro-, Post- and Synbiotics

It is now widely accepted that the human microbiota plays a fundamental role in health and disease, and an increasing number of studies have been conducted for the clarification of the possible uses of interventional strategies using substances that act at different “stages” of the human microbiota. Collectively, these strategies compose the family of biotics: prebiotics, probiotics, postbiotics, and synbiotics, each play distinct and complementary roles in maintaining intestinal health [16].

Prebiotics are selectively utilized non-digestible dietary components (e.g., inulin, fructo-oligosaccharides, galacto-oligosaccharides) that stimulate the growth and activity of beneficial gut bacteria, particularly Bifidobacterium and Lactobacillus. Their fermentation leads to increased production of SCFAs, which are implicated in the maintenance of epithelial barrier integrity, modulation of immune responses, as well as support of metabolic health.

Probiotics are live microorganisms (most commonly Lactobacillus and Bifidobacterium species) that, when administered in adequate amounts, directly modulate the gut microbiota, by inhibiting pathogen colonization, strengthening the mucosal barrier, and regulating local and systemic immune responses. They possess strain-specific effects and promote eubiosis.

Postbiotics are preparations of inactivated microorganisms as well as their metabolites (such as SCFAs, peptides, and cell wall components) that confer health benefits without requiring live bacteria. Postbiotics can reinforce gut barrier function, reduce inflammation, and exert antimicrobial effects.

Synbiotics are combinations of prebiotics and probiotics designed to synergistically enhance the survival and activity of beneficial microbes. Synbiotics can improve the viability of probiotics, further promote SCFA production, and more effectively restore or maintain a balanced gut microbiota, with evidence supporting benefits for immune modulation, metabolic health, and cognitive function.

Collectively, these biotics help maintain microbial diversity, support epithelial integrity, regulate immune and metabolic functions, and protect against dysbiosis and related health disturbances.

### 1.4. Objectives of the Study

It is demonstrated that dysbiosis in pediatric patients with SBS plays a central role in their clinical outcomes. Children with SBS exhibit markedly reduced gut microbial diversity, with a predominance of potentially pathogenic taxa such as Enterobacteriaceae and Proteobacteria, and a depletion of beneficial commensals including Firmicutes and Bacteroidetes. This altered microbiota composition is associated with increased risks of SIBO, intestinal inflammation, impaired intestinal adaptation, and complications such as CRBSIs, liver injury and poor linear growth. Dysbiosis also contributes to metabolic disturbances, including reduced production of SCFAs and altered bile acid metabolism, which further impair nutrient absorption and mucosal health [17].

This narrative review aims to critically assess the existing body of evidence concerning the use of pre-, pro- and synbiotics in the management of pediatric SBS and related intestinal dysbiosis, in addition to identifying the unmet needs in this line of research, in order to pave the way for future investigations to allow the expansion of the current evidence base.

## 2. Materials and Methods

A structured literature search was conducted in PubMed, covering studies published from 2001 to November 2025.

Gray literature sources, including conference proceedings and trial registries, were not considered to ensure the inclusion of only peer-reviewed, high-quality evidence.

Search terms included: short bowel syndrome, intestinal failure, pediatric, child, prebiotics, probiotics, synbiotics, postbiotics, and microbiota.

The initial search retrieved 32 articles. After screening titles and abstracts, one redundant article was removed.

The eligibility criteria (PICO) of all articles were manually reviewed to answer the following research question: ‘Are pre-, pro-, post- and synbiotics safe and effective in the management of pediatric SBS?’

The specific criteria were:

- Population: pediatric patients with postsurgical SBS (neonates, children and adolescents up to 18 years of age);
- Interventions/Exposures: microbiota features/measurements and pre-, pro-, post- or synbiotic treatments;
- Comparators: absence of microbiota-targeted interventions.

Following the full-text assessment, 16 records were excluded because they did not meet these eligibility criteria (e.g., studies involving adult populations, studies focusing on other microbiota-targeted interventions, or studies lacking relevant clinical or microbiological data). One additional study was discarded on the basis of the non-availability of its full text. The search also retrieved three systematic reviews, which were manually analyzed by the reviewers, and the relevant literature identified was included in the analysis.

**Figure 1:**
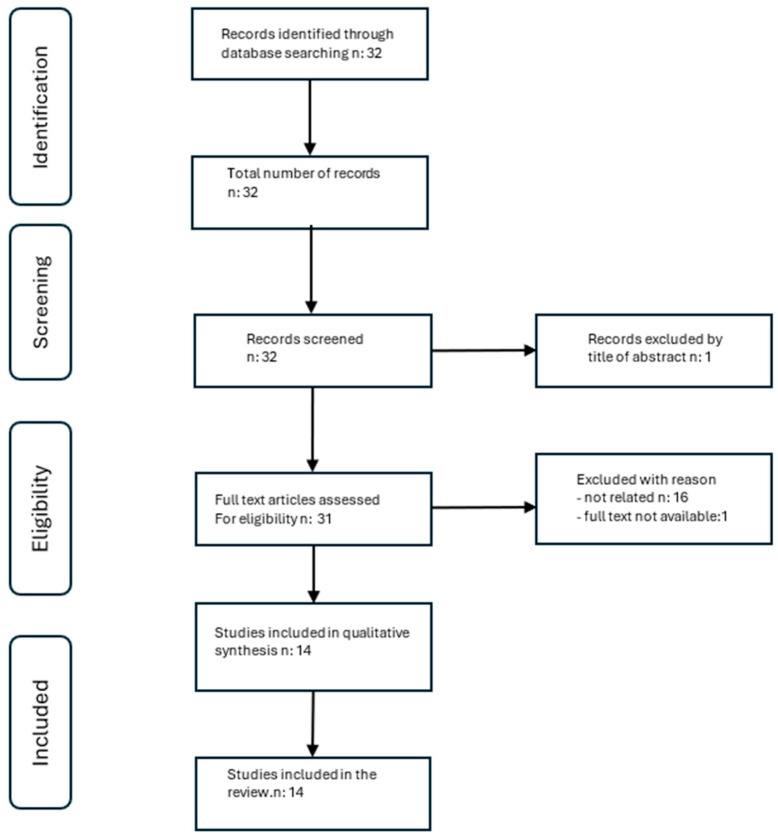
PRISMA flow diagram.

Therefore, 14 articles were included in the review (Figure 1).

## 3. Results

A total of 14 articles were selected for full inclusion in this narrative review. These comprised:

Four clinical studies (observational cohorts, interventional trials) (Table 1).

Four preclinical/translational animal studies (Table 2).

Six case reports relevant to microbiota-targeted interventions in pediatric SBS (Table 3).

Three reviewers independently reviewed the titles and abstracts of the identified studies to determine their relevance to the research topic.

Case reports were included in the qualitative synthesis to provide evidence on rare complications and potential benefits in specific clinical scenarios, given the scarcity of randomized controlled trials.

The objective of the present review was to determine whether microbiota-targeted treatments are safe and effective in reducing intestinal dysbiosis and related symptoms in pediatric SBS patients.

Outcome measures were the following:

(1) Safety: Trend of adverse events following treatment.
(2) Efficacy: Trend of clinical symptoms attributable to dysbiosis before and after treatment.

### 3.1. Probiotics

The largest body of evidence concerning the use of biotics in children with SBS regards the use of probiotics. First evidence originates from animal models. In an experimental study involving rats undergoing 80% small bowel resection, authors found that supplementation with *Bifidobacterium lactis* significantly reduced bacterial translocation (BT) from 87% to 50% of subjects, suggesting a protective effect on the intestinal barrier and a decreased risk of sepsis [18]. Similar findings were reported in another murine massive resection model (75%), where administration of a probiotic mixture reduced BT to mesenteric lymph nodes, liver and systemic circulation, while also improving mucosal parameters, with evidence of reduced enterocyte apoptosis and increased crypt depth in the ileum [19]. Collectively, these preclinical data established the rationale that probiotics may play a dual role in both limiting dysbiosis and promoting structural adaptation of the remaining intestine.

However, more robust evidence from controlled trials has been less encouraging. In a double-blind, placebo-controlled cross-over study involving nine children with SBS matched to twelve healthy controls, four weeks of *Lactobacillus rhamnosus* GG (LGG) supplementation did not result in significant improvements in intestinal permeability, nor in stable colonization by the probiotic strain. Throughout both treatment phases, the investigators measured intestinal permeability using the lactulose/mannitol ratio, performed quantitative fecal cultures to assess Lactobacillus colonization, and obtained hydrogen breath tests (HBT) to evaluate small intestinal bacterial overgrowth. During LGG supplementation, fecal colonization by Lactobacillus did not increase consistently compared with placebo, indicating limited engraftment of the probiotic strain. Likewise, LGG administration did not produce consistent changes in intestinal permeability, with no significant differences in lactulose/mannitol ratios between treatment and placebo periods. An isolated finding was observed in a single SBS patient, in whom LGG therapy coincided with a shift to a positive HBT, suggesting a possible alteration in bacterial growth dynamics [20].

More recently, a randomized trial published in 2020 assessed the efficacy of LGG and *Lactobacillus johnsonii* administration over two months in 18 children weaned from PN but showing suboptimal growth. No significant differences compared with placebo were observed in either fecal microbiota composition or anthropometric outcomes, suggesting that probiotic supplementation with standard Lactobacillus strains may be insufficient in clinically stable patients [5].

Parallel preclinical research has further clarified microbiological and metabolic mechanisms. In a neonatal piglet model with 75% small bowel resection, probiotic supplementation (Lactobacilli and Bifidobacteria) increased microbial diversity and butyrate production, whereas empiric metronidazole therapy reduced diversity and expanded the proportion of Proteobacteria and Enterobacteriaceae. These findings underscore the detrimental impact of antibiotic-driven dysbiosis and the potential of probiotics to restore a more favorable, eubiotic state [21].

Finally, recent translational studies have identified novel molecular pathways. LGG supplementation was shown to activate intestinal farnesoid X receptor (FXR) signaling and the downstream FGF15/19 pathway, leading to upregulation of occluding (a tight junction transmembrane protein, involved in maintenance of epithelial barriers), improved mucosal morphology (increased villus height, crypt depth, and goblet cell numbers), reduction in Proteobacteria, and enrichment of beneficial secondary bile acids. These effects were abolished in intestinal FXR knockout models, confirming an FXR-dependent mechanism [22].

Such findings suggest that probiotics may exert their effects not only through microbiota modulation, but also by acting as functional “post-biotics,” capable of driving intestinal adaptation via specific host signaling pathways.

### 3.2. Synbiotics

The first clinical attempts of synbiotic therapy implementation in children date back to 2004, when Kanaomori et al. investigated the effects of long-term synbiotic therapy administered for more than one year in seven patients with short bowel syndrome complicated with refractory enterocolitis. The intervention combined *Bifidobacterium breve*, *Lactobacillus casei*, and the prebiotic galacto-oligosaccharides. Notable improvements were reported: the intestinal microbiota shifted toward a more favorable composition, with increased levels of beneficial anaerobes such as Bifidobacteria, Lactobacilli, and other non-pathogenic obligate anaerobes involved in SCFA production, alongside a reduction in potentially pathogenic bacteria. These microbial changes were accompanied by a substantial rise in fecal SCFA concentrations, which nearly doubled over the treatment period. Clinically, most patients appeared to benefit from the treatment: six of the seven children exhibited accelerated weight gain, and five showed increases in serum rapid-turnover proteins, suggesting an improvement in overall nutritional status [23].

Similar results were obtained by Uchida et al., in their small cohort of pediatric patients with SBS who received a synbiotic combination consisting of *Bifidobacterium breve*, *Lactobacillus casei* and galacto-oligosaccharides for at least 12 months. Treatment was shown to be associated with increased fecal SCFA production, improved immunonutritional parameters, as indicated by increased peripheral lymphocyte counts and prealbumin, and a favorable trend in growth. Microbiological analyses confirmed enrichment of fecal flora with Bifidobacteria and Lactobacilli [24].

No study concerning the isolated use of prebiotics or postbiotics was identified during our search.

In addition to clinical trials, several case reports have documented the use of probiotics and synbiotics in pediatric patients with SBS or SBS-related complications. In one case, a child with only 25 cm of residual small intestine and severe dysbiosis, a synbiotic combination (*Bifidobacterium breve*, *Lactobacillus casei*, galacto-oligosaccharides) rapidly restored a healthier anaerobic flora, reduced pathogenic overgrowth, and led to resolution of infections, metabolic instability, and feeding intolerance, ultimately enabling transition off parenteral nutrition [25].

A second report described an infant with 60 cm of residual jejunum and persistent high-output diarrhea despite optimized medical therapy; introduction of *Lactobacillus casei* resulted in measurable intestinal colonization, improved sodium balance, reduced stool frequency, and successful weaning from parenteral support [26].

Lastly, Yilmaz et al. reported a child with recurrent D-lactic acidosis driven by overgrowth of D-lactate-producing Lactobacillus. Probiotics containing non-D-lactate-producing Bifidobacterium strains effectively suppressed the offending organisms and normalized D-lactate levels, resolving correlated neurological symptoms [27].

Analysis of case reports also allowed evaluation of possible adverse effects of treatment with biotics in children with SBS.

De Groote et al. (2005) reported a clinical case of an 11-month-old female with SBS who developed bacteremia after five weeks of administration of LGG via a gastrostomy tube [28]. Similarly, Kunz et al. described two cases of children with SBS who developed Lactobacillus bacteremia during treatment with probiotics containing LGG. Both patients had severe impairment of intestinal integrity and were dependent on parenteral nutrition. Blood cultures were positive for Lactobacillus species, and molecular analyses of the blood isolates and the administered probiotics demonstrated genetic identity, indicating the probiotic as the likely source of the infection [29]. In addition to systemic infection, probiotics have been implicated in D-lactic acidosis in patients with extensive intestinal resection. Munakata et al. reported a five-year-old girl with SBS who developed acute ataxia and high anion gap metabolic acidosis while receiving Lactomin, a carbohydrate-fermenting probiotic. Serum D-lactic acid levels were markedly elevated, and cessation of the probiotic, along with targeted antimicrobial therapy led to resolution of metabolic abnormalities and clinical symptoms [30].

**Table 1:** Clinical studies selected for review and their characteristics.

Date	Reference	Study Model and Population	Intervention	Effect on Microbiota Composition	Clinical Outcomes	Adverse Events
2020	[5]	Pilot RCT, 18 children with SBS	Probiotics (*Lactobacillus rhamnosus*; *Lactobacillus johnsonii*)	No significant change in the fecal microbiota.	No observed effects on growth.	No adverse event reported.
2008	[20]	Crossover RCT, 9 children with SBS	Probiotics (*Lactobacillus rhamnosus* GG)	No significant difference in the rate of colonization by Lactobacilli between the two groups.	No significant effect.	Possible SIBO.
2007	[24]	Prospective study, 4 children with SBS	Synbiotics (*Bifidobacterium breve*; *Lactobacillus casei*; galacto-oligosaccharides)	Normalization of fecal flora post-treatment (similar to healthy controls).	Increased production of fecal SCFA, improved laboratory markers (prealbumin, lymphocytes) improved growth curve.	No adverse event reported.
2004	[23]	“Prospective case series”, 7 children with SBS with refractory enterocolitis	Synbiotics (*Bifidobacterium breve*; *Lactobacillus casei*; galacto-oligosaccharides	Normalization of fecal microbiota (dominance of Bifidobacter; drastic decrease in *Candida*, *E. coli*, *Pseudomonas*, MRSA).	Increased production of fecal SCFA. Increased weight gain; improved nutritional status.	No adverse event reported.

**Table 2:** Preclinical animal studies selected for review and their characteristics.

Date	Reference	Study Model and Population	Intervention	Effect on Microbiota Composition	Clinical Outcomes	Adverse Events
2007	[19]	Murine models with 75% bowel resection.	Probiotics (*Lactobacillus* GG)	Not described.	Improved gut barrier function and adaptation (significant reduction in bacterial translocation to lymph nodes; intestinal regrowth).	No adverse event reported.
2022	[21]	Piglet models with 75% bowel resection.	Probiotics (*Lactobacillus*; *Bifidobacterium*) vs. Metronidazole	Increased alpha-diversity in probiotics group compared to the other two, with increased butyrate production.	Not described.	No adverse event reported.
2025	[22]	Translational study (murine models + children with SBS)	Probiotics (*Lactobacillus rhamnosus* GG)	Decreased abundance of Proteobacteria.	Improved intestinal morphology; enhanced gut barrier integrity.	Not described.
2002	[18]	Murine models with 80% bowel resection.	Probiotics (*Bifidobacterium lactis*)	Decreased bacterial translocation.	Improved weight gain.	Not described.

**Table 3:** Case reports included in the qualitative analysis and their characteristics.

Date	Reference	Study Population	Intervention	Effect on Microbiota Composition	Clinical Outcomes	Adverse Events
2001	[26]	Child with SBS, 12 months.	Probiotics (*Lactobacillus casei*)	Abundant Lactobacilli at day 3 (previously absent).	Decreased stool frequency; improved Na^+^ balance; eventual discontinuation of PN.	No adverse event reported.
2005	[28]	Child with SBS, 11 months.	Probiotics (*Lactobacillus rhamnosus*)	Not described.	Not described.	Post-treatment bacteremia from Lactobacilli.
2004	[29]	Two children with SBS, 1 month and three months old.	Probiotics (*Lactobacillus* GG)	Not described.	Not described.	Post-treatment bacteremia from Lactobacilli.
2001	[25]	Child with SBS.	Synbiotics (*Bifidobacterium breve*; *Lactobacillus casei*; galactoolisaccharides)	Pre-treatment: abundant *E. coli* and *Candida*. Post-treatment: decreased abundance in favor of Lactobacilli and Bifidobacter;	Decreased events of acidosis; decreased stool frequency; improved weight gain; increased production of fecal SCFA.	No adverse event reported.
2018	[27]	Case report, child with SBS (18 months).	Probiotics (non-D-lactate-producing cocktail: *L. rhamnosus* GG; *B. lactis*; *B. breve*; *B. longum*)	Disappearance of D-lactate-producing *L. plantarum*; increased alpha-diversity and species richness.	Prevention of recurrent D-lactic acidosis; clinical stability without need for antibiotics for >1 year.	No adverse event reported.
2010	[30]	Case report, child with SBS (5 years).	Probiotics (Lactomin: containing *L. acidophilus*, *L. bulgaricus*, *S. faecalis*, *S. faecium*)	Not directly sequenced but inferred overgrowth of D-lactate- producing bacteria (Lactobacillus).	Development of acute ataxia and metabolic acidosis (D-lactic acidosis). Symptoms resolved after stopping probiotics.	D-lactic acid encephalopathy (D-lactic acidosis).

## 4. Discussion

The transition from theoretical promise to clinical application of the family of biotics in pediatric SBS, as evidenced by the analyzed studies, is characterized by a delicate balance between risk and benefit. On one hand, the safety analysis underscores the vulnerability of this population, particularly regarding septic complications; on the other, the nutritional outcomes observed in specific cohorts suggest that restoring intestinal homeostasis can tangibly impact growth trajectories. To contextualize these conflicting results, it is essential to examine how the modulation of dysbiosis varies according to the disease stage.

The analysis of the literature reveals that dysbiosis in pediatric SBS is not a static condition, but a dynamic spectrum heavily influenced by the mode of nutritional support. As detailed in Table 4, patients dependent on PN exhibit a distinct microbiological signature characterized by “starvation” of the microbiome. The lack of luminal nutrients selects against beneficial obligate anaerobes (e.g., Bacteroidetes, Firmicutes) that require fermentable fibers, leading to a loss of colonization resistance. This ecological void is rapidly filled by opportunistic facultative anaerobes, particularly Proteobacteria and Enterobacteriaceae, as demonstrated in both the piglet models by Pauline et al. [21] and clinical cohorts by Kanamori et al. [23] The clinical consequence of this PN-associated dysbiosis is primarily infectious: the bloom of Gram-negative pathogens, combined with increased mucosal permeability, creates a high-risk environment for bacterial translocation and catheter-related sepsis.

Conversely, as patients progress towards weaning from PN, thus transitioning to enteral autonomy, the risk profile shifts from infectious to metabolic. While bacterial diversity improves, the introduction of high carbohydrate loads to a shortened bowel with altered motility can precipitate the overgrowth of specific fermenting organisms. Notably, Yilmaz et al. [27] and Munakata et al. [30] highlighted that in weaned patients, an overabundance of D-lactate-producing Lactobacillus species (such as *L. plantarum* or *L. delbrueckii*) can ferment malabsorbed carbohydrates into D-lactate. Since humans lack the enzyme D-2-hydroxyacid dehydrogenase to metabolize this isomer efficiently, it accumulates, leading to D-lactic acidosis and encephalopathy. Therefore, we postulate that therapeutic approaches should be tailored, with PN-dependent patients possibly benefiting from synbiotics aimed at restoring anaerobes with the aim of preventing risk of sepsis, while weaned patients require careful monitoring of Lactobacillus strains to avoid metabolic toxicity, and may benefit from favoring non-D-lactate-producing probiotics like Bifidobacteria.

Based on this microbiological dichotomy, the safety profile must be interpreted with caution.

Infectious and metabolic complications in pediatric SBS are highly relevant to achieving intestinal autonomy and represent the main causes of morbidity and mortality in these patients, often prolonging dependence on PN and hindering intestinal adaptation, which is fundamental for acquiring enteral autonomy. Such complications may interfere with this process by increasing the risk of systemic infections, worsening liver function, and impeding growth and development, resulting in a prolonged need for PN [31,32].

Although the theoretical rationale for using probiotics in SBS is to restore eubiosis and improve barrier function, the safety profile in this specific population remains a critical concern. In our cumulative analysis of approximately 50 pediatric patients across the included studies and case reports, four serious adverse events (8%) were recorded, highlighting that these interventions are not devoid of risk. These complications can be categorized into two distinct pathophysiological entities: systemic infections and metabolic acidosis. They are elucidated in Table 4.

The most frequently reported severe complication was Lactobacillus bacteremia, which accounted for three out of the four serious adverse events.

De Groote et al. reported sepsis in an 11-month-old infant after 5 weeks of LGG administration via gastrostomy [28]. Similarly, Kunz et al. [29] described two cases of Lactobacillus bacteremia in children treated with LGG. Molecular analysis of the blood isolates and the administered probiotic product confirmed genetic identity, proving the probiotic was the source of infection. A crucial pattern emerges from these reports: all cases of bacteremia occurred in patients who were dependent on PN and CVC carriers. No systemic infections were reported in weaned patients without central access.

The mechanism is likely to involve bacterial translocation across a compromised intestinal barrier (common in SBS) or direct contamination of the CVC. The increased intestinal permeability in SBS could allow live probiotic bacteria to breach the mucosa and reach the systemic circulation; in the presence of a foreign body such as a CVC, these bacteria could colonize the device and cause persistent sepsis.

The second major safety signal is D-lactic acidosis, observed in one patient within the analyzed cohort.

Munakata et al. [30] described a 5-year-old girl with SBS who developed acute ataxia and high anion gap metabolic acidosis while receiving Lactomin, a carbohydrate-fermenting probiotic.

This complication is unique to SBS anatomy: short bowel syndrome often leads to carbohydrate malabsorption and stasis. When patients consume carbohydrate-rich meals alongside probiotics that are prolific D-lactate producers (such as certain Lactobacillus species), the bacteria ferment the unabsorbed sugars into D-lactate. Unlike L-lactate, the human body metabolizes D-lactate poorly; its accumulation leads to metabolic acidosis and possibly to neurotoxicity.

In the reported case, cessation of the probiotic and administration of antibiotics led to the resolution of symptoms, confirming the proposed etiology.

Based on this safety analysis, we may suggest some key points to address in clinical practice, which should also be included in future research. Strict monitoring in CVC holders is advisable, as the risk of translocation-induced sepsis appears to be higher in patients with central lines and severe mucosal injury. The use of live microorganisms in this subgroup requires extreme caution

Moreover, in SBS patients, we need to mitigate the metabolic risks due to the use of probiotic strains capable of producing D-lactate; this could be reached, possibly favoring the choice of non-D-lactate-producing strains, such as Bifidobacterium species or specific Lactobacillus strains validated as safe. Indeed, it is important to note that the ability to produce D-lactate is highly strain-specific and not universal across species. For example, Lactobacillus reuteri and Lactobacillus plantarum can produce significant amounts of D-lactate, while Lactobacillus helveticus is more associated with L-lactate production [33].

On the other hand, Bifidobacterium species do not produce D-lactate and might be recommended for vulnerable groups, and there is evidence that switching to Bifidobacterium-based probiotics can even help normalize D-lactate levels by suppressing the offending flora [27], albeit the available literature in this regard is quite scarce to date. Postbiotic alternatives, such as heat-treated (non-viable) strains of Lactobacillus and Bifidobacterium, as well as purified SCFA formulations, might be more suitable for such fragile patients, as they do not colonize the gut or produce D-lactate, and might promote bifidogenic effects [3].

When analyzing the impact of microbiota modulation on auxological parameters, a dichotomy emerges based on the duration of the intervention and the type of treatment employed. Short-term studies utilizing probiotics alone failed to demonstrate significant growth improvements. Specifically, Sentongo et al. [20] found that a 4-week course of LGG in children with SBS did not result in measurable anthropometric changes. Similarly, in a more recent randomized trial by Piper et al. [5], a 2-month supplementation with *Lactobacillus rhamnosus* and *Lactobacillus johnsonii* in eighteen stable patients weaned from PN showed no significant difference in weight or length z-scores compared to placebo.

In stark contrast, studies employing long-term synbiotic regimens (combining probiotics with prebiotics for >12 months) have reported substantial benefits. Kanamori et al. [23] observed that six out of seven patients (85%) with refractory enterocolitis exhibited accelerated weight gain and improved nutritional status (as measured by an increase in rapid-turnover proteins) after more than a year of treatment with *Bifidobacterium breve*, *Lactobacillus casei* and galacto-oligosaccharides. Corroborating these findings, Uchida et al. [24] reported a favorable trend in both height and weight gain velocity, alongside significant increases in prealbumin levels, in a cohort treated with a similar symbiotic mixture for at least 12 months. These data strongly suggest that the trophic effects of microbiota modulation, likely mediated by sustained SCFA production and mucosal adaptation, require a prolonged timeframe to translate into tangible improvement in growth.

Moreover, a physiological lag time may be expected between the microbiological correction and auxological improvement; thus, short-term studies (4–8 weeks) likely capture the initial microbiological shifts but are insufficient to observe the final somatic outcome.

It is noteworthy that the trials registering positive effects on auxological parameters were not merely longer but utilized symbiotic rather than probiotics alone. We postulate that the addition of prebiotics may be crucial in SBS to support the engraftment of beneficial strains in a hostile intestinal environment, thereby enabling the sustained metabolic effects necessary for growth.

Furthermore, the lack of effect in the Piper cohort might also reflect a “plateau” in clinically stable, PN-weaned patients, whereas subjects suffering from refractory inflammation and malabsorption, had a greater margin for nutritional recovery and so-called catch-up growth once dysbiosis was corrected, suggesting that microbiota modulation may be more effective in improving growth in patients with active failure or inflammation rather than in stable, weaned patients.

Beyond study duration and patient clinical status, the route and formulation of probiotic administration were variably reported across the included literature, encompassing oral powders, liquid suspensions, and enteral tube administration. This heterogeneity limits direct comparison of efficacy and safety outcomes, as the delivery matrix and administration route can significantly influence the viability of probiotic strains in the hostile gastrointestinal environment and their subsequent biological activity. Future clinical trials should aim for standardized reporting of these parameters to better understand their clinical impact.

The potential positive impact of microbiota modulation on growth parameters, observed particularly in symbiotic trials, can be mechanistically explained through several pathophysiological pathways highlighted by preclinical models. The restoration of a eubiotic microbiota enhances energy harvest through the production of SCFAs: as demonstrated in a neonatal piglet model of massive resection [21], probiotic supplementation (Lactobacilli and Bifidobacteria) significantly increased microbial diversity and butyrate production compared to antibiotic treatment. This biochemical shift was confirmed in the clinical setting [23], with evidence of doubling fecal SCFA concentrations, which correlated directly with accelerated weight gain and improved serum protein levels. Since SCFAs provide a crucial energy source for colonocytes and can contribute significantly to the host’s daily energy requirements, their depletion in SBS dysbiosis represents a loss of energy that biotic therapy aims to recover.

Moreover, experimental evidence suggests that specific probiotic strains actively promote mucosal adaptation, thereby increasing the absorptive surface area. In rat models of massive resection, supplementation with *Bifidobacterium lactis* [18] and probiotic mixtures [19] not only reduced bacterial translocation but also improved mucosal architecture by increasing crypt depth and reducing enterocyte apoptosis. Molecular mechanisms for this trophic effect have more recently been elucidated: LGG was shown to activate the intestinal FXR pathway, leading to upregulation of tight junction proteins and increased villus height [22].

Therefore, the observed clinical growth improvement is likely the composite result of two synergistic mechanisms: the decontamination of the small bowel from Gram-negative pathobionts, such as Proteobacteria, leading to a reduction in mucosal inflammation and caloric consumption due to infectious stress, and the direct stimulation of epithelial proliferation, which maximizes the absorptive capacity of the remnant bowel.

## 5. Conclusions

In conclusion, the current body of evidence suggests that pre-, pro-, and symbiotic hold significant theoretical potential to mitigate dysbiosis and support intestinal adaptation in pediatric SBS. However, the translation of this potential into clinical practice is currently limited by the heterogeneity of study designs and legitimate safety concerns. Our analysis underscores that the risks of bacteraemia and metabolic acidosis, although rare, are specific to identifiable subgroups, namely, catheter-dependent patients.

It is necessary to acknowledge some limitations that warrant caution in the interpretation of the findings. Firstly, the scarcity of available evidence is a primary constraint: the analysis relies on a limited number of studies characterized by marked heterogeneity in design, ranging from preclinical models to anecdotal case reports. The lack of large-scale, multicenter randomized controlled trials limits the statistical power and the generalizability of the data regarding efficacy.

Moreover, the interpretation of microbiota modulation is complicated by confounding factors, most notably the concurrent use of antibiotics. Given the high susceptibility of SBS patients to catheter-related sepsis, frequent antibiotic courses may independently drive dysbiosis or mask the potential benefits of biotic interventions, making it difficult to isolate the net effect of the treatment.

Finally, the variability in probiotic strains, dosages, and treatment durations employed across the included studies currently hinders the definition of a standardized therapeutic protocol.

Future research perspectives should include more rigorous randomized control studies with well-defined endpoints (for example, autonomy from PN, infection rates, IFALD progression), longer duration of therapy, and careful safety monitoring. Advances in microbiome research also open the possibility of targeted approaches, including precision probiotics, defined consortia, tailored prebiotics, and postbiotic metabolites that may deliver benefits without the risks of live organisms. Large-scale longitudinal studies aiming to clarify how microbiome signatures predict intestinal rehabilitation are already ongoing, such as the MIRACLe protocol [34].

Until such targeted therapies are validated, the use of biotics in pediatric SBS should be reserved for selected cases, where the balance between promoting growth and preserving safety can be meticulously monitored.

## Figures and Tables

**Table 4:** Differential microbiota signatures and clinical risks in pediatric SBS: impact of nutritional status.

Microbiota	Alteration in PN-Dependent SBS	Alteration in Off-PN SBS	Clinical Consequences
Bacterial diversity	Severely depleted: lack of enteral nutrients leads to “starvation” of commensals.	Reduced but recovering; improving richness, but still distinct from healthy controls.	Reduced colonization resistance; susceptibility to pathogen invasion and overgrowth
Dominant strains	Proteobacteria.	Firmicutes/Bacteroidetes recovery.	PN: pro-inflammatory state, mucosal injury; Off-PN: altered fermentation.
Specific pathogens	Enterobacteriaceae (e.g., *E. coli*, *Klebsiella*)	Risk of D-lactate-producing Lactobacillus strains overgrowth.	PN: translocation, central line-associated bloodstream infections, IFALD
Metabolic Activity	Minimal SCFA production (low butyrate and acetate due to lack of substrate)	High fermentation activity.	PN: mucosal atrophy. Off-PN: D-lactic acidosis.

## Data Availability

No new data were created or analyzed in this study.

## Abbreviations

The following abbreviations are used in this manuscript:

SBS	short bowel syndrome
PN	parenteral nutrition
SCFA	short-chain fatty acids
CVC	central venous catheter
IFALD	intestinal failure-associated liver disease
CRBI	catheter-related bloodstream infection
SIBO	small intestinal bacterial overgrowth
BT	bacterial translocation
LGG	*Lactobacillus rhamnosus* GG
HBT	hydrogen breath test
FXR	farnesoid X receptor
